# Supplementary material for: Is the Triggering of PD-L1 Dimerization a Potential Mechanism for Food-Derived Small Molecules in Cancer Immunotherapy? A Study by Molecular Dynamics
Source: Int J Mol Sci. 2023 Jan 11;24(2):1413. doi: 10.3390/ijms24021413 (PMC9864258; doi:10.3390/ijms24021413)
Supplement: Supplementary file 1 [file ijms-24-01413-s001.zip › ijms-2103521-supplementary.pdf]

Is the triggering of PD-L1 dimerization a potential mechanism for food-derived small molecules in cancer immunotherapy? A study by molecular dynamics

Xiaoyan Wu, Na Wang, Jianhuai Liang, Bingfeng Wang, Yulong Jin, Boping Liu* and Yang Yang *

Key laboratory for Bio-Based Materials and Energy of Ministry of Education, College of Materials and Energy, South China Agricultural University, Guangzhou 510630, China; wawdtam123@163.com (X.W.); wangna1020@foxmail.com (N.W.); j.h_liang@stu.scau.edu.cn (J.L.); wbfeng@scau.edu.cn (B.W.); jyl@scau.edu.cn (Y.J.)

* Correspondence: boping@scau.edu.cn (B.L.); yyang@scau.edu.cn (Y.Y.)

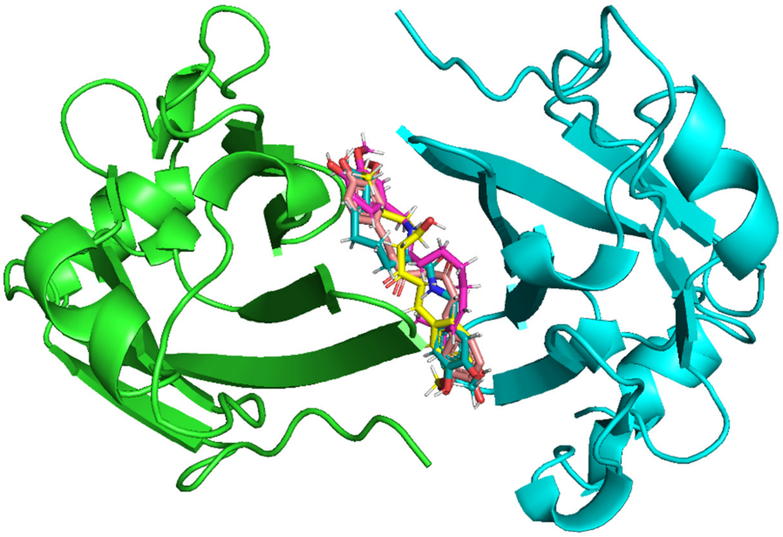

Figure S1. Initial structures of the systems used in MD simulations. initial structure of capsaicin, zucapsaicin, curcumin and 6-gingerol are cyan, purple, brown and yellow, respectively. The green chain and blue chain correspond to (A)PD-L1 and (B)PD-L1.

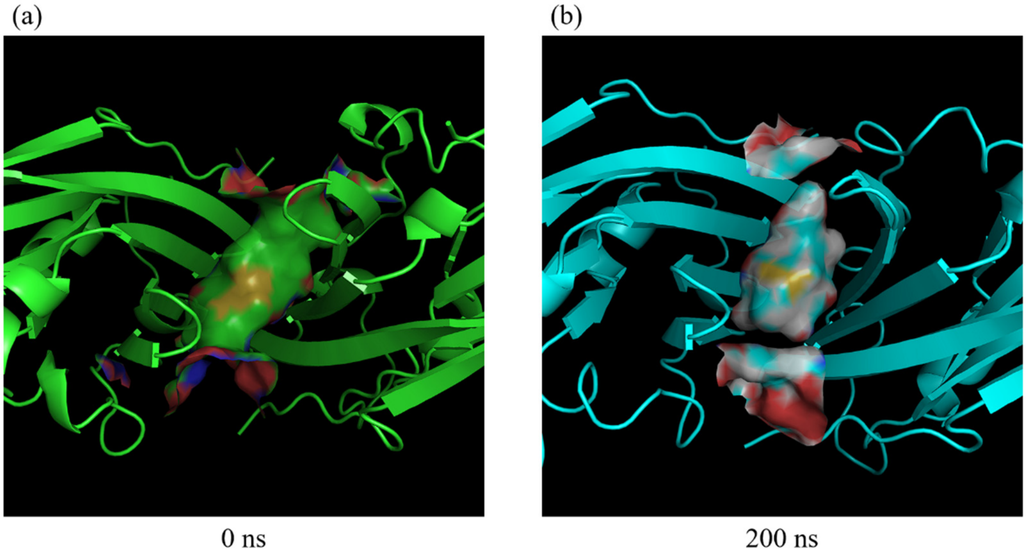

Figure S2. PD-L1 dimer without small molecules in MD simulations. (**a**) The opened binding pocket and PD-L1 dimer are marked as green in initial stage of MD simulation. (**b**) The closed binding pocket and PD-L1 dimer are marked as blue in final stage of MD simulation.

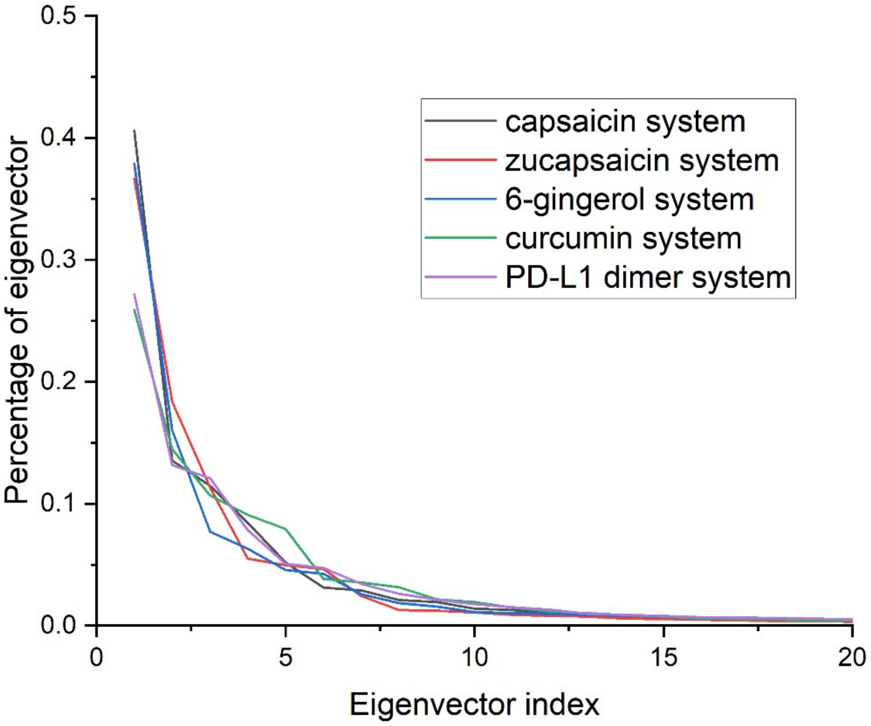

Figure S3. The percentage of top 20 eigenvalues of covariance matrix (C_α) of capsaicin, zucapsaicin, 6-gingerol and curcumin system in MD simulations.

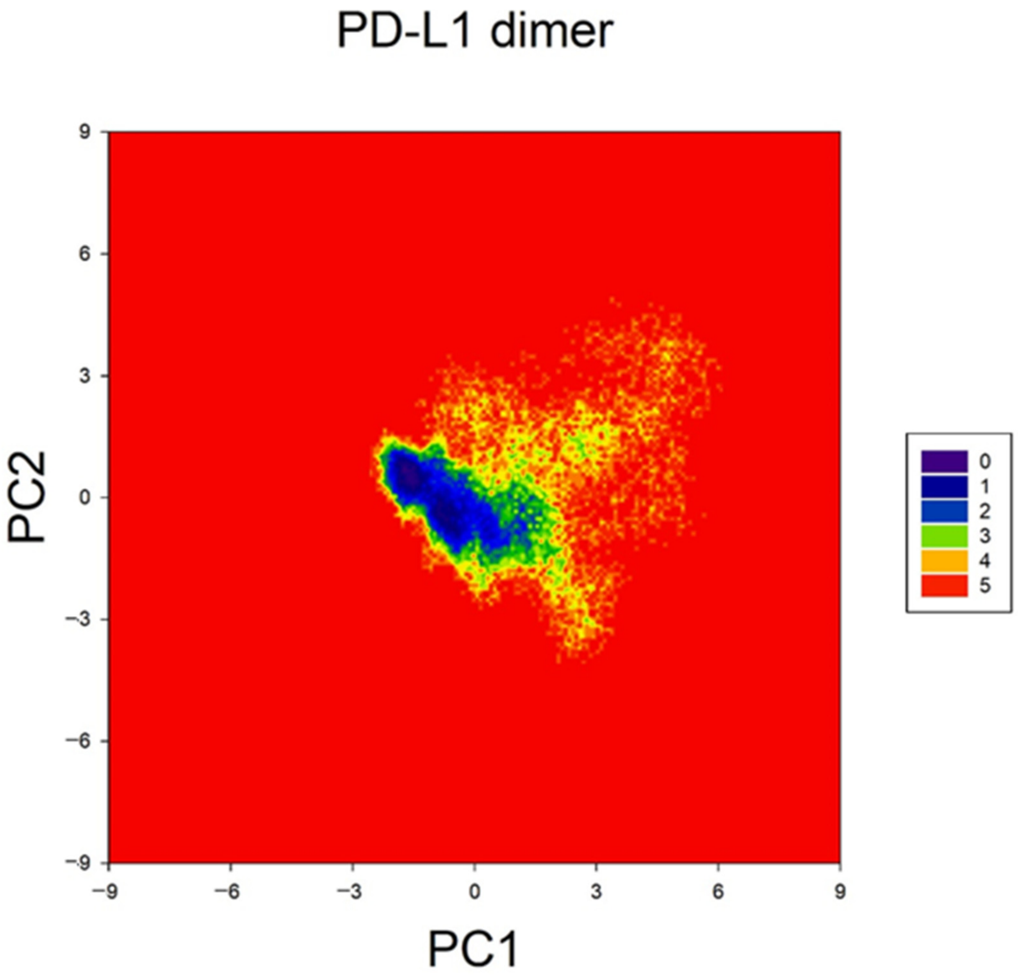

Figure S4. The free energy landscape (FEL) of dimer system (C_{α}) in 200ns MD simulations.

Table S1. The Binding free energy and Mindist of PD-L1 of two chains((A)PD-L1 and (B)PD-L1).

	Capsaicin	Zucapsaicin	6-gingerol	curcumin
Mindist of (A)PD-L1	115.96	79.53	135.12	122.81
Mindist of (B)PD-L1	131.23	119.29	149.05	87.89
Binding free enegy of (A)PD-L1	−7.857 kcal/mol	−7.177 kcal/mol	−9.281 kcal/mol	−10.261 kcal/mol
Binding free enegy of (B)PD-L1	−11.485 kcal/mol	−11.179 kcal/mol	−10.805 kcal/mol	−8.482 kcal/mol

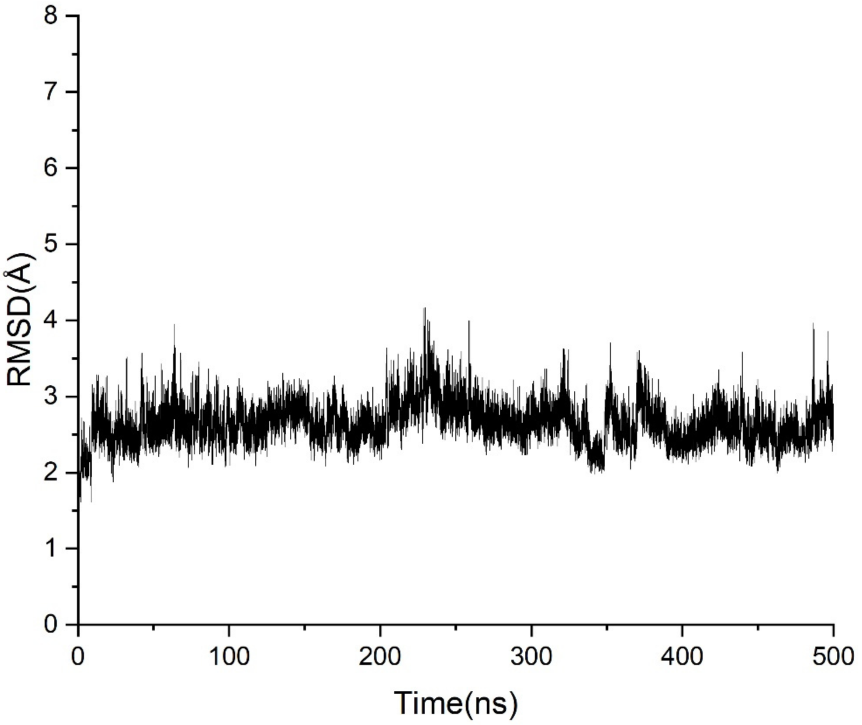

Figure S5. The RMSD of PD-L1 dimer in curcumin system in 500ns simulation.

As shown in Figure S5, the results showed that the complex system reached equilibrium state at 10ns and kept this state for the subsequent 490ns simulation. Considering that the data produced by 200ns simulation is enough for all systems to reach equilibrium and longer simulation time would consume too much computing resources, all the complex systems in this study were simulated by 200ns and repeated for three times.

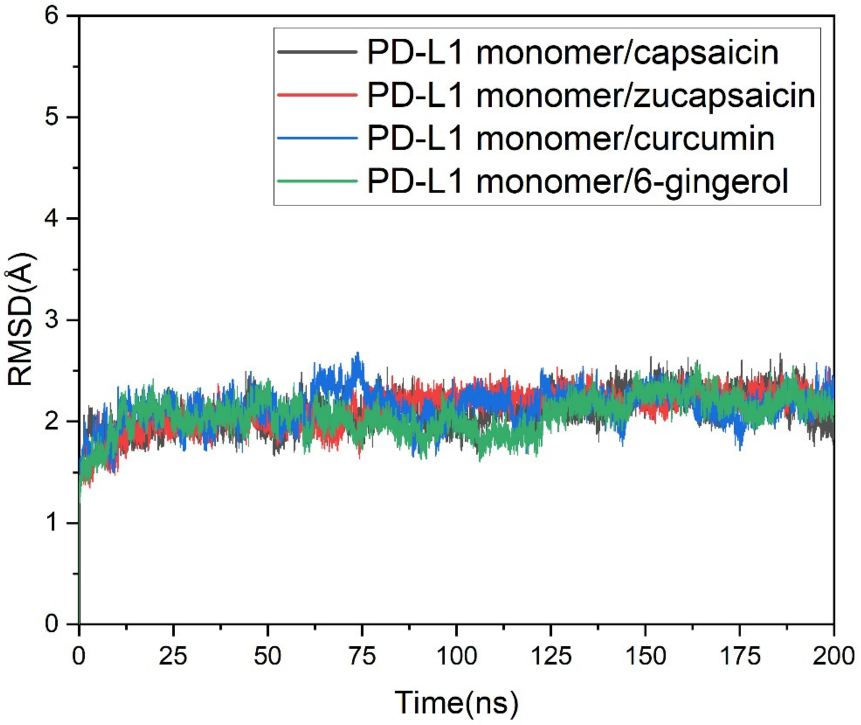

Figure S6. The RMSD of PD-L1 monomer/capsaicin, PD-L1 monomer/zucapsaicin, PD-L1 monomer/curcumin, PD-L1 monomer/6-gingerol system in 200ns simulation.
